# Blood glucose variability in patients with acute pancreatitis

**DOI:** 10.1002/ueg2.12224

**Published:** 2022-03-19

**Authors:** Yuzuru Ohshiro

**Affiliations:** ^1^ Department of Internal Medicine Omoromachi Medical Center Naha City Okinawa Japan

**Keywords:** acute pancreatitis, diabets, epidemiology, pancreas, screening

I have read the original article by Bharmal et al. regarding blood glucose variability (GV) during the course of acute pancreatitis with great interest.^1^ Although it has been shown that around 23% of patients with acute pancreatitis develop diabetes mellitus within 3 years discharge from hospital,^2^, ^3^ the pathophysiology of acute pancreatitis associated diabetes is poorly understood. However, Bharmal et al. identified increased GV during the course of acute pancreatitis as a risk factor of developing diabetes. The study has several limitations. First, patients in the subgroup with high GV developed diabetes later; but the possibility that the group had already included some patients with pre‐existing diabetes should be considered. The researchers excluded preexisting diabetes by the criteria including admission level and history of elevated HbA1c. The primary etiologies of acute pancreatitis in patients in their study included alcoholism and biliary. Such patients, especially those with alcoholism, occasionally experience periods of decreased food intake and malnutrition, leading to decreased levels of HbA1c on admission. This makes it difficult to exclude pre‐existing diabetes by admission level of blood glucose or HbA1c. Second, hyperglycemia is often observed during the course of acute pancreatitis.^4^ To assess GV, it is critical to analyze different infusion fluids (infusion rate of glucose) during treatment, and whether to use antihyperglycemic agents including insulin. Thus, a treatment protocol is needed to assess GV among. Third, it has been well shown that severe pancreatitis, including necrotic pancreatitis, can cause islet loss leading to diabetes, partly due to a damaging pancreatic issue. Further, the strong association of severity and subsequent development of diabetes has been reported.^4^ This study, however, did not seem to find an association of severity of pancreatitis with the development of diabetes.

As mentioned, the pathophysiology of acute pancreatitis associated diabetes is multifactorial and poorly understood. However, this study is important for providing a strong predictor of diabetes developing after suffering acute pancreatitis.

## Data Availability

No data.
